# Myopia prevalence in Malaysian children: a systematic review and meta-analysis

**DOI:** 10.3389/fpubh.2026.1836591

**Published:** 2026-06-25

**Authors:** Chee Fai Sui, Chin Hui Ng, Yee Chang Soh, Kah Seng Lee, Serena Leow, Qi Ying Lean, Jack Bee Chook, Long Chiau Ming

**Affiliations:** 1 Faculty of Medical and Life Sciences, Sunway University, Sunway City, Malaysia; 2 School of Pharmaceutical Sciences, Universiti Sains Malaysia, Minden, Malaysia; 3 Department of Pharmacy, Hospital Raja Permaisuri Bainun, Ipoh, Malaysia; 4 PPD, Inc., Thermo Fisher Scientific, Kuala Lumpur, Malaysia; 5 Faculty of Arts and Social Sciences, Sunway University, Sunway City, Malaysia; 6 Faculty of Pharmacy, Universiti Teknologi MARA, Cawangan Pulau Pinang, Kampus Bertam, Kepala Batas, Malaysia

**Keywords:** child health, epidemiology, good health and wellbeing, prevalence, vision disorders

## Abstract

**Introduction:**

Myopia is a growing global concern, projected to affect nearly half the world’s population by 2050. East and Southeast Asia face a disproportionate burden, with early-onset cases leading to severe complications in children. In Malaysia, epidemiological data on childhood myopia remain limited and inconsistent, hindering accurate burden estimation.

**Aim:**

To systematically review and synthesize the prevalence of myopia among Malaysian children aged 4–18 years, providing national and subgroup-specific estimates.

**Methodology:**

A PRISMA-compliant search was conducted across five databases. Eligible studies included cross-sectional, cohort, or population-based designs published in English. Two reviewers independently screened and extracted data. A random-effects meta-analysis using the Freeman–Tukey double arcsine transformation pooled prevalence rates from 17 studies. Subgroup and sensitivity analyses explored heterogeneity by age, ethnicity, region, cycloplegia status, and diagnostic method. Publication bias was assessed via funnel plots, Egger’s, Begg’s, and trim-and-fill methods. Study quality was appraised using the JBI checklist.

**Results:**

Of 1,147 records, 21 studies were included in the synthesis, with 17 suitable for meta-analysis (*n =* 19,281). The unadjusted pooled prevalence of myopia was 25.0% (95% CI: 15.0–37.0%), with substantial heterogeneity (I^2^ = 99.3%). Subgroup analysis showed that studies using non-cycloplegic refraction yielded a higher prevalence estimate than studies using cycloplegic refraction (31% versus approximately 16%). The trim-and-fill adjusted prevalence was 15.8%, suggesting that the unadjusted pooled estimate may overestimate clinically confirmed myopia prevalence because of non-standardised measurement methods, variable diagnostic thresholds ranging from ≤ − 0.25 D to ≤ − 1.00 D, and possible small-study or publication effects. Higher prevalence was also observed in older studies, smaller samples, and subjective methods, and increased with age and among Chinese children. Eight studies were high quality, eight moderate, and one low.

**Conclusion:**

Childhood myopia represents an important public health concern in Malaysia. However, the magnitude of prevalence should be interpreted with caution because of high heterogeneity, variation in myopia definitions, and the predominance of non-cycloplegic measurement methods, all of which may cause the unadjusted estimate to overestimate clinically confirmed prevalence. Although the unadjusted pooled prevalence was 25.0%, the trim-and-fill adjusted estimate of 15.8% and the cycloplegic subgroup estimate of approximately 16% suggest that the clinically confirmed prevalence is likely to lie closer to the lower end of an approximate range of 16 to 25%. Future nationally representative studies using standardised cycloplegic refraction and consistent diagnostic criteria are needed to guide resource allocation, screening, and policy planning.

**Systematic review registration:**

https://www.crd.york.ac.uk/PROSPERO/view/CRD420251012425, CRD420251012425.

## Highlights

- The unadjusted pooled prevalence of myopia among Malaysian children aged 4–18 years was 25.0% (95% CI: 15.0%–37.0%); however, after trim-and-fill adjustment for small-study effects the estimate was 15.8%, and the cycloplegic subgroup estimate was approximately 16%. The clinically confirmed prevalence is therefore likely to lie within an approximate range of 16% to 25%, and the unadjusted 25.0% figure should be interpreted with caution rather than as a definitive national estimate.
- Estimates are highly dependent on measurement technique; non-cycloplegic methods reported a significantly higher prevalence (31%) than studies using the gold standard cycloplegic refraction (16%). This highlights that non-standardized methods tend to overestimate the true burden due to residual accommodation.
- Prevalence increases sharply with age, escalating from 1.0% in preschool cohorts to 30% in primary school children, correlating with school years and near work. Rates are highest among Chinese children (42.4%–64.6%) and lowest in the Orang Asli population (5.5%–9.3%).
- To account for potential reporting bias, the initial pooled prevalence of 25.0% was adjusted using trim-and-fill analysis, yielding a lower, more scientifically defensible estimate of 15.8%. The current evidence suggests the true burden lies in the range of 16%–25%.
- Myopia represents an emerging public health challenge in Malaysia, occupying an intermediate position between high-prevalence East Asian countries and lower-prevalence Western nations

## 1 Introduction

Myopia is a growing global health concern, with projections indicating that nearly half of the world’s population will be affected by 2050 (1). The burden is particularly severe in East and Southeast Asia, where early-onset myopia in children often progresses rapidly, increasing the risk of high myopia and its associated complications such as retinal detachment, myopic maculopathy, and glaucoma (2, 3). In school-aged populations, uncorrected myopia is already a leading cause of visual impairment, significantly impacting educational performance, psychosocial development, and long-term ocular health (4).

Despite increasing attention to this issue, the epidemiological landscape of childhood myopia in Malaysia remains poorly defined. One of the earliest studies described myopia in Malaysian primary one child was published in 1982, but it provided limited methodological details and did not report spherical equivalent refraction (SER) cut-off points, making interpretation and comparison difficult (5). In contrast to countries such as China (6), Singapore (3), and South Korea (7), where large-scale studies have produced robust prevalence data, Malaysian evidence is limited, fragmented, and methodologically inconsistent. Existing studies vary in sampling strategies, case definitions, and measurement techniques, with most being small-scale or school-based surveys (8). These limitations hinder comparability, reduce generalizability, and often fail to capture Malaysia’s rich ethnic and geographic diversity (9). Critically, no comprehensive systematic review or meta-analysis has synthesized available data, leaving a significant gap in understanding the true burden and distribution of childhood myopia in the Malaysian context.

This study aims to address this gap by systematically reviewing and synthesizing the prevalence of myopia among Malaysian children aged 4–18 years. By pooling data across studies, we will generate national and subgroup-specific prevalence estimates stratified by age, ethnicity, geographic region, and measurement method. This study will bring together scattered findings from previous research to give a more complete picture of how common myopia is among children in Malaysia. The results can assist clinician make better decisions, guide public health efforts to prevent and manage myopia, and support the development of policies that address this growing issue.

## 2 Methodology

### 2.1 Search strategy

This review was conducted in accordance with the Preferred Reporting Items for Systematic Reviews and Meta-Analyses (PRISMA) framework (10). The protocol was developed following the PRISMA-P 2015 checklist (11) and registered in the International Prospective Register of Systematic Reviews (PROSPERO) under reference number CRD420251012425.

A comprehensive literature search was performed to identify studies reporting the prevalence of myopia among children aged 4 to 18 years in Malaysia. The initial search was conducted using PubMed, with the strategy adapted to suit the syntax and indexing of additional databases, including Scopus, the Cochrane Library, and ProQuest. Keywords and MeSH terms related to “myopia,” “children,” “prevalence,” and “Malaysia” were combined using Boolean operators to refine the search.

An additional search was carried out using Google Scholar through the Publish or Perish software. This tool retrieves and analyzes academic citations from sources like Google Scholar, enabling researchers to calculate bibliometric indicators and identify relevant literature that may not be indexed in traditional databases. Due to the broad string-matching approach used by Google Scholar, the search was limited to the first 1,000 results to ensure relevance and manageability. Search strategy is included in Appendix 1.

### 2.2 Eligibility criteria

Studies were selected according to predefined inclusion and exclusion criteria to ensure relevance and data specificity. Eligible studies comprised populations of children aged 4 to 18 years diagnosed with myopia. Study design: Cross-sectional, cohort, or population-based studies.

Studies reporting other refractive conditions were excluded unless they provided stratified data specific to myopia within the targeted age range. Only articles published in English were considered, reflecting a limitation imposed by the language proficiency of the author. For studies involving broader age groups, inclusion was contingent upon clear age-specific data breakdowns relevant to children aged 4–18 years.

Exclusion criteria encompassed studies involving children younger than 4 years, investigations focused on non-myopic refractive errors such as hyperopia or astigmatism, and article such as review articles, conference proceedings, and abstracts.

### 2.3 Study selection

All identified records were imported into a reference management software Endnote 21, and duplicates were removed. Titles and abstracts were screened independently by two reviewers using Rayyan software [SCF and NCH). Full texts of potentially eligible studies were retrieved and assessed for inclusion based on the predefined criteria in 2.2. Discrepancies were resolved through discussion or consultation with a third reviewer (LCM).

For the studies that met the inclusion criteria, further identification was carried out to determine the availability of data suitable for meta-analysis. Only studies that provided sufficient quantitative information such as prevalence rates, sample sizes, and measurement methods—were included in the pooled analysis. Studies that lacked the necessary data for statistical synthesis were instead incorporated into a broader narrative synthesis, which aimed to summarize and contextualize findings across the literature.

### 2.4 Data management and data extraction

Following the screening process, data extraction was conducted independently by two reviewers [SCF and NCH] using a standardized Excel template. This template was designed to systematically capture key characteristics of each study, including the names of the authors and year of publication, study design and setting, sample size and age range of participants, definition and method of myopia measurement, reported prevalence rates, geographic location within Malaysia, and the ethnic composition of the study population. Ethnicity was categorized as either specific; where the study focused on a single ethnic group or mixed, where multiple ethnic groups were represented.

### 2.5 Statistical analysis

#### 2.5.1 Outcome measure

A meta-analysis was conducted to estimate the pooled prevalence of myopia among Malaysian children aged 4 to 18 years. To account for variability across studies, a random-effects model was applied, reflecting anticipated heterogeneity in study populations and methodologies. Prevalence rates were reported as proportions, each accompanied by a 95% confidence interval (CI) to reflect statistical uncertainty. To stabilize variance and accommodate extreme proportions, prevalence estimates were transformed using the Freeman-Tukey double arcsine method and subsequently back-transformed for interpretability (12).

#### 2.5.2 Subgroup and sensitivity analysis

Heterogeneity across studies was assessed using the I^2^ statistic, with values exceeding 50% considered indicative of substantial heterogeneity. To explore sources of heterogeneity and assess the robustness of pooled prevalence estimates, we conducted both subgroup and sensitivity analyses. Subgroup analyses were performed based on key study-level characteristics hypothesized to influence myopia prevalence, including age range, ethnicity, region, year of publication, cycloplegia status, diagnostic method, and study quality ranking. These variables were selected *a priori* based on biological plausibility and prior evidence suggesting differential prevalence across these strata. Sensitivity analyses were conducted to evaluate the stability of the findings by excluding studies with small sample sizes, varying diopter thresholds, and lower methodological quality. Additionally, we assessed the impact of cycloplegia status and extreme prevalence values on the overall estimates. These analyses aimed to ensure that the pooled results were not influenced by specific study features or methodological decisions.

#### 2.5.3 Publication bias

Publication bias was assessed through visual inspection of funnel plots and formally tested using Egger’s regression method. To further support the findings, additional tests including the trim-and-fill method and Begg’s rank correlation test were conducted. All statistical analyses were performed using R software (R version 4.4.1) with computations carried out by SCF.

### 2.6 Quality assessment and rating

The methodological quality and risk of bias of the included studies were assessed independently by two reviewers using the Joanna Briggs Institute (JBI) Critical Appraisal Checklist for Studies Reporting Prevalence Data. This tool evaluates key domains such as sampling strategy, sample size adequacy, measurement reliability, and statistical analysis. Each item was rated as “Yes,” “No,” “Unclear,” or “Not Applicable,” and discrepancies were resolved through discussion or consultation with a third reviewer. The results of the quality assessment were used to inform the interpretation of findings and to explore potential sources of heterogeneity in the meta-analysis.

For each item in the quality assessment checklist, we assigned one point for a “Yes” response, while “No” and “Unclear” responses received no points. Based on the total score, studies were categorized into three quality levels: Low quality (1–3 “Yes” responses), Moderate quality (4–6 “Yes” responses), and High quality (7–9 “Yes” responses). This scoring system enabled a structured comparison of study quality and helped ensure that our synthesis reflects the reliability of the underlying evidence.

## 3 Results

### 3.1 Narrative synthesis

#### 3.1.1 Study selection and characteristics

**Figure 1:**
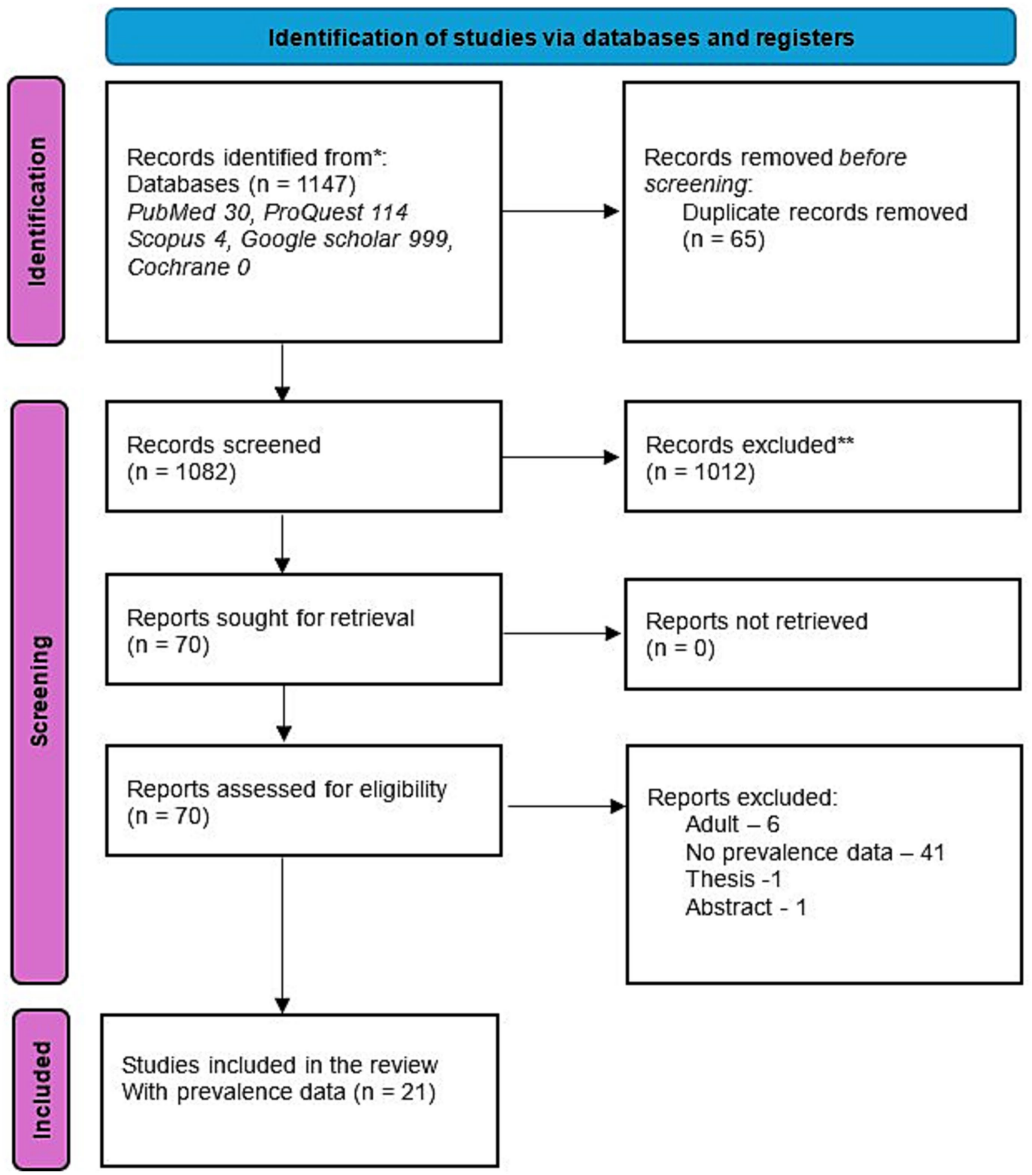
PRISMA flowchart.

A total of 1,147 records were identified across five databases: PubMed (30), ProQuest (114), Scopus (4), and Google Scholar (999), with no records retrieved from Cochrane (Figure 1). After removing 65 duplicates, 1,082 records were screened. Of these, 1,012 were excluded based on title and abstract. Seventy full-text articles were assessed, and 49 were excluded due to adult populations (6), lack of prevalence data (41), thesis format (1), or abstract-only publication (1). Ultimately, 21 studies met the inclusion criteria.

The total sample size across the 21 included studies was 21,903 children (8, 9, 13–31). However, two studies with sample sizes of 1,752 (25) and 870 (26) were subset analyses derived from a larger trial involving 4,634 participants (8). To avoid duplication in estimating the overall sample size, these subset studies were considered part of the original cohort.

After accounting for this overlap, the adjusted total sample size is 19,281 children (8, 9, 13–25, 27–31).

To facilitate interpretation of the representativeness of the data, studies were grouped by sample size. Most studies had moderate sample sizes ranging from 100 to 999 participants (8, 9, 13, 15–24, 29–31), while four studies included between 1,000 and 4,999 children (8, 16, 17, 30). Four studies had small sample sizes of fewer than 100 participants (13, 18, 20, 22), and only one study had a large sample size exceeding 5,000 (14). Characteristics of studies reporting the prevalence of myopia among school-age children in Malaysia is presented in Table 1.

**Table 1:** Characteristics of studies reporting the prevalence of myopia among school-age children in Malaysia.

First author (year of publication)	Region	Age group (yr)	Sample size	Ethnicity	Cycloplegia	Refraction assessment	Prevalence of myopia (%)	Myopia cases	Notes
Garner F. L. et al. (1990) (29)	PM	6–17	904	Specific^1^	No	Objective	28	253	
Chung KM et al. (1996) (30)	PM	6–20	1883	Specific^2^	No	Objective	42.4	794	
Mohidin and Lee (2009) (27)	PM	7–18	749	Specific^3^	No	Objective	16R/17.6 L	120R/132 L	Exclude MA
Goh PP et al. (2005) (8)	PM	7–15	4,634	Mixed	Yes	Objective	19.3	895	
Saw SM et al. (2006)^ (41)	PM	7–9	1752	Mixed	Yes	Objective	13.4	235	Exclude MA
Hashim SE et al. (2008) (31)	PM	6–12	705	Specific^1^	No	Objective	5.4	38.0	
Farhana AB et al. (2012) (9)	EM	7,12,15	293	Specific*	Yes	Objective	45.1	132.0	
Farhana AB et al. (2012)* (9)	PM	6–12	60	Mixed	No	Subjective	61.7	37	
Ramlee and Goh (2012)^ (26)	PM	7–8	870	Mixed	Yes	Objective	10.1	88.0	Exclude MA
MZ Nurulain et al. (2012) (14)	PM	7–17	5,200	Mixed	NR	NR	7.02	365	
Premsenthil M. et al. (2013) (28)	EM	4–6	400	Mixed	Yes	Objective	3R/3.5 L	12R/14 L	Exclude MA
Jayaraman et al. (2016) (15)	NR	10–12	168	Specific^2^	NR	NR	58.4	98	
Chew et al. (2018) (16)	PM	4–6	1,287	Mixed	Yes	Objective	1.01	13	
Madhavan (2018) (17)	PM	7–11	1,462	Specific^3^	No	Objective	28.9	422.0	
Omar R. et al. (2019) (19)	PM	7–12	110	Specific^4^	Yes	Objective	5.5	6.0	
Fairuz et al. (2020) (20)	PM	4–12	43	Specific^4^	Yes	Objective	9.3	4	
Leng et al. (2021) (21)	EM	7–15	569	Mixed	No	Objective	14.6	83	
Isa et al., (2021) (22)	PM	7–17	19	NR	No	Subjective	57.9	11	
Omar R et al. (2022) (18)	PM	7–12	82	Specific^2^	No	Subjective	64.63	53	
Ismail and Sukumaran (2022) (23)	PM	8–12	245	NR	Yes	Objective	30.2	74.0	
Wardati HJ et al. (2024) (24)	PM	7–12	480	Specific^1^	No	Objective	7.1	34	

PM, Peninsular Malaysia; EM, East Malaysia. ^Data is subset from Goh PP et al., NR -not reported, Malay^1^, Chinese^2^, Indian^3^, Orang asli^4^, R-right eye, L-Left eye, MA -Meta-analysis.

#### 3.1.2 Prevalence of myopia

Reported prevalence rates ranged from 1.01% (16) to 64.63% (18) among Malaysian schoolchildren, with notable variation by region, ethnicity, and diagnostic method. In Peninsular Malaysia, prevalence ranged from 5.4% (31) to 61.7% (13) while East Malaysia ranged from 3.0% (28) to 45.1% (9). Urban cohorts and Chinese ethnicity were associated with higher prevalence (15, 18, 30).

Cycloplegic refraction, used in six studies (8, 9, 16, 19, 20, 23), generally yielded lower prevalence estimates compared to non-cycloplegic methods (13, 17, 18, 21, 22, 24, 29–31).

**Figure 2:**
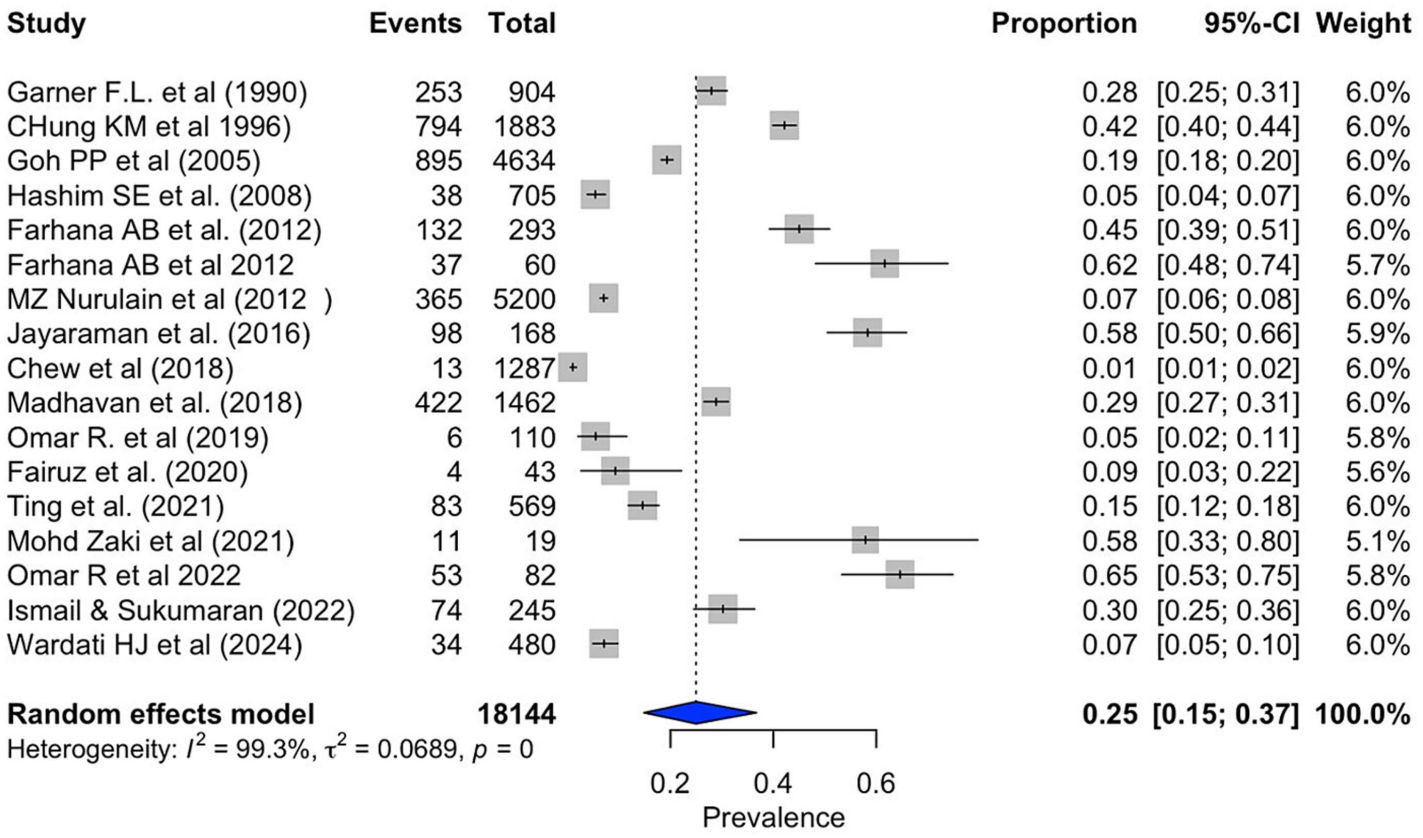
Forest plot of the included studies.

More recent studies (2020–2024) reported narrower prevalence ranges (7.1–30.2%) compare with early 90s (28.0–42.4%) suggesting methodological refinement or stabilization. Two studies (9.5%) were published before 2005 (29, 30), 9 studies (38.1%) were published between 2005 and 2015 (8, 9, 13, 14, 25–28, 31), and 10 studies (52.4%) were published from 2016 onwards (15–24). This distribution indicates an increasing number of publications over time (Figure 2).

Two studies reported eye-specific prevalence, consistently showing slightly higher rates in the left eye by Mohidin et al. (27): 17.6% L vs. 16% R and Premsenthil et al. (28): 3.5% L vs. 3.0% R.

#### 3.1.3 Methodological and population variability

Diagnostic approaches varied: six studies used cycloplegic refraction (gold standard) (8, 9, 16, 19, 20, 23), 10 used non-cycloplegic methods (13, 17, 18, 21, 22, 24, 29–31), one relied on optometrist-led diagnosis (14), and one used medical record (15). Cut-off criteria for myopia also differed: 12 studies used ≤ − 0.50 D (8, 9, 13, 17, 19, 20, 22–24, 26, 27, 29), one used −0.25 D (18), two ≤ − 1.00 D (17, 28), and four did not specify (14–16, 21).

Ethnic composition varied across studies: nine focused on specific groups (Malay, Chinese, Indian, Orang Asli), eight included mixed populations, and two did not report ethnicity (22, 23). Geographically, three studies were conducted in East Malaysia (Sarawak) (9, 21, 28), while 16 were in Peninsular Malaysia (8, 13–20, 24–27, 29–31), including seven in Kuala Lumpur and others across Johor, Selangor, Kelantan, Pahang, and Negeri Sembilan. Ethnicity-specific breakdown revealed notable disparities in myopia prevalence. The Orang Asli population showed the lowest prevalence, ranging from 5.5 to 9.3% (*n =* 2) (19, 20), while Chinese children exhibited the highest rates, ranging from 42.4 to 64.6% (*n =* 3) (15, 18, 30). Indian children had a reported prevalence of 28.9% (*n =* 1) (17), and Malay children ranged from 5.4 to 28.0% (*n =* 3) (24, 29, 31).

Meanwhile, the age group ranges spanned from early childhood to late adolescence. Three studies included younger cohorts starting age 4 (16, 28, 29), and one study extended to age 20 (30). Variability in age brackets (e.g., 10–12 years vs. 7–17 years) reflects differences in study design and may influence prevalence estimates.

### 3.2 Meta-analysis of the included studies

#### 3.2.1 Pooled prevalence

Seventeen studies were included in the meta-analysis, focusing on the prevalence of myopia among Malaysian children aged 4–18 years. Due to substantial heterogeneity across studies (I^2^ = 99.3%), a random-effects model was employed. The pooled prevalence of myopia was estimated at 0.25 (95% CI, 0.15–0.37), indicating that approximately one in four children were affected. The high heterogeneity suggests variability in study design, population characteristics, or measurement methods, warranting further investigation through subgroup analyses.

#### 3.2.2 Subgroup analysis and sensitivity analysis

To explore the sources of heterogeneity, subgroup analyses were conducted based on several study characteristics. These analyses revealed significant variations in pooled prevalence across different subgroups, as detailed in Table 2. Studies conducted before 2005 consistently reported higher prevalence rates compared to more recent studies. Similarly, a clear inverse relationship was observed between sample size and prevalence, with smaller studies showing a considerably higher pooled prevalence. The diagnostic method used also emerged as a critical factor, with studies employing a subjective method reporting a substantially higher prevalence than those using an objective approach. Furthermore, studies that did not use a cycloplegic agent found a higher prevalence of myopia. Across education levels, prevalence varied widely, with the highest rates found in studies of mixed education levels and primary school children, and the lowest in preschool cohorts. Finally, studies with specific ethnic populations showed a higher pooled prevalence compared to those with mixed ethnic groups.

**Table 2:** Subgroup and sensitivity analysis of the included studies.

Subgroup	No. studies	Prevalence	95% CI	*P*
Year				0.52
<2005	2	0.35	0.22,0.49	
2005–2015	5	0.24	0.07,0.48	
≥2015	10	0.24	0.10,0.41	
Sample size^S^				0.18
≥4,000	2	0.13	0.03,0.27	
1,000–2000	3	0.20	0.01,0.53	
500–999	3	0.15	0.04,0.30	
<500	9	0.35	0.18,0.54	
Education				<0.05
All Level	2	0.35	0.22,0.49	
Primary-Secondary	5	0.25	0.10,0.45	
Primary	6	0.3	0.11,0.52	
Preschool-Primary	3	0.21	0.0,0.61	
Preschool	1	0.01	0.0.0.02	
Ethnicity ^NR = 2^				0.33
Specific	10	0.27	0.14,0.42	
Mixed	5	0.16	0.02,0.39	
NR	2	0.41	0.17,0.69	
Cycloplegic ^NR = 2^				0.35
Yes	6	0.16	0.05,0.31	
No	9	0.31	0.16,0.48	
Method ^NR = 2^				<0.05
Objective	12	0.17	0.10,0.27	
Subjective	3	0.63	0.55,0.70	
Region ^NR = 1^				<0.05
Peninsular Malaysia	14	0.22	0.12,0.35	
East Malaysia	2	0.29	0.05,0.61	
Sensitivity				
High-Moderate Quality	16	0.23	0.13,0.35	
Large Sample Size (≥500)	8	0.16	0.07,0.27	
Standard Se ≤ −0.5d	11	0.25	0.14,0.39	
Pool prevalence				
Random Effect	17	0.25	0.15,0.37	
Fixed Effect	17	0.16	0.15,0.16	

P, subgroup differences; S, no study with sample size between 2000 and 4,000, ^NR^ Study with no data reported.

Sensitivity analysis was performed by re-calculating the pooled prevalence based on different criteria. Excluding studies with sample sizes <500, the pooled prevalence was 16% (95% CI, 7 to 27%). Similarly, the pooled prevalence from high-moderate quality studies was 23% (95% CI, 13 to 35%). The analysis of studies published after 2015 resulted in a pooled prevalence of 24% (95% CI, 10 to 24%). These results suggest that the overall pooled estimate may be sensitive to specific study characteristics. Forest plot subgroup is presented in Appendix 2.

#### 3.2.3 Publication bias

**Figure 3:**
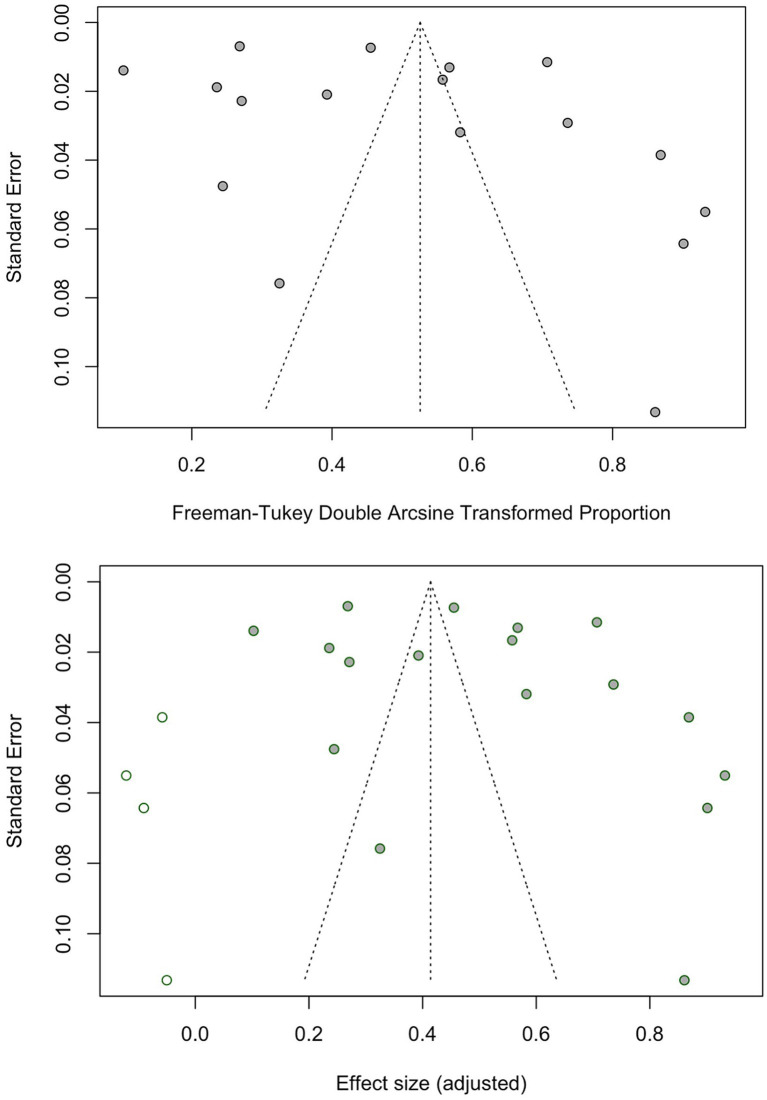
Funnel plot publication bias (top) and trim and fill plot (bottom).

Visual inspection of the funnel plot revealed mild asymmetry, characterized by a relative absence of smaller studies reporting lower prevalence estimates, which could indicate selective publication of studies with higher prevalence rates or significant findings. Egger’s linear regression test (t = 1.18, df = 15, *p* = 0.257) and Begg’s rank correlation test (z = 0.25, *p* = 0.805) were both conducted, with neither showing statistically significant evidence of publication bias. However, the high heterogeneity observed in our analysis (I^2^ = 99.3%, τ^2^ = 0.1127) may have reduced the sensitivity of these tests (Figure 3).

To account for potential missing studies, a trim-and-fill analysis was performed, which imputed four additional studies to adjust for the funnel plot asymmetry. The initial pooled prevalence of the 17 studies was 0.25 (95% CI 0.15, 0.37). After the imputation, the adjusted prevalence was 0.1583 (95% CI 0.0661, 0.2791), suggesting that publication bias may have had a greater influence on the overall effect size than initially thought. The robustness of our findings is supported by the consistency across multiple assessment methods, though the interpretation remains tempered by the substantial heterogeneity inherent in the included studies.

#### 3.2.4 Quality assessment and risk of bias

As part of the quality assessment, all 17 included studies were evaluated using the JBI Prevalence Critical Appraisal Checklist, which consists of nine items assessing methodological rigor and reporting transparency. This approach is consistent with methodological guidance for systematic reviews of observational epidemiological studies reporting prevalence and cumulative incidence data, which recommends that critical appraisal and evidence synthesis be specifically tailored to the design and reporting features of observational prevalence studies, and that pooled prevalence estimates from heterogeneous observational data be interpreted with appropriate caution (32).

Among the included studies, eight were rank as High quality, eight as Moderate quality, and one as Low quality. A consistent strength across studies was the clear description of study subjects and settings, with nearly all articles meeting this criterion. However, common weaknesses were observed in the reporting of statistical analysis and response rate management, where many studies either failed to meet the criteria or provided insufficient information. Other domains such as sample frame appropriateness, sampling methods, sample size adequacy, coverage during analysis, and use of valid measurement tools—showed mixed performance, indicating variability in methodological rigor across studies. JBI assessment table is presented in Appendix 3.

## 4 Discussion

### 4.1 Summary of findings

Our pooled analysis of 17 studies showed an unadjusted myopia prevalence of 25.0% among Malaysian children aged 4–18 years. This estimate is comparable with a broader Asian meta-analysis reporting a pooled prevalence of 24.2% among individuals younger than 20 years (33), and with WHO Western Pacific Region reports indicating a Malaysian prevalence of 34.4% (34). Similarly, a recent Malaysian systematic review by Tan et al. (35) reported a pooled prevalence of 17.18% among 13,367 children and adolescents, with prevalence increasing by age from 2.54% in children aged 0 to 6 years, to 26.48% at 7–12 years, and 42.71% at 13 to 18 years. Consistent with this age-related pattern, our findings also showed a sharp increase in prevalence from 1.0% in preschool cohorts to 30% among primary school children, suggesting that myopia burden escalates with school age, likely reflecting increased educational demands and near-work exposure. This variation supports a cautious interpretation of the current findings and suggests that differences in age range, ethnic composition, study scope, and refraction method may partly explain variation across estimates.

After accounting for potential small-study and publication bias effects using the trim-and-fill method, the adjusted prevalence decreased from 25.0 to 15.8%. This adjusted estimate closely aligns with the cycloplegic subgroup estimate of approximately 16%, suggesting that the clinically confirmed burden may be closer to one in six children. Therefore, rather than interpreting the unadjusted 25.0% estimate as a definitive national prevalence, the current evidence is more appropriately understood as supporting a prevalence range of approximately 16 to 25%.

The very high heterogeneity observed in this meta-analysis (I^2^ = 99.3%) further supports this cautious interpretation. Important sources of heterogeneity include refraction method, participant age, study setting, sampling frame, geographical location, and diagnostic cut-off, which ranged from ≤ − 0.25 D to ≤ − 1.00 D. Such variation can affect case classification and limits direct comparability across studies. Accordingly, the pooled prevalence should be viewed as an indicative synthesis of heterogeneous Malaysian studies rather than a precise national estimate derived from uniform diagnostic criteria. This distinction has important implications for public health planning. Treating the unadjusted 25.0% estimate as definitive may overestimate the number of children with clinically confirmed myopia and inflate projections for screening, clinical workload, optical correction, and budget allocation. Conversely, the unadjusted estimate remains useful as an indicator of the broader screening-detected burden in the available literature. A tiered interpretation is therefore warranted: the unadjusted estimate reflects the current heterogeneous evidence base, whereas the trim-and-fill adjusted estimate of 15.8% and the cycloplegic subgroup estimate of approximately 16% provide a more conservative basis for estimating clinically confirmed prevalence.

Subgroup analyses revealed notable methodological variation. Studies that did not employ cycloplegia reported a higher prevalence than those that did (31% versus 16%). This disparity is consistent with the tendency of non-cycloplegic refraction to overestimate myopia because of residual accommodation, particularly in paediatric populations (36).

These findings support the recommendation by Morgan et al. that cycloplegic refraction should be used as the gold standard in epidemiological research (37). Similarly, Wilson et al. reported that non-cycloplegic refraction tends to overestimate myopia and underestimate hyperopia compared with cycloplegic refraction and concluded that cycloplegic refraction remains the preferred method for measuring refractive error in children aged ≤12 years (38). This supports the interpretation that the higher non-cycloplegic subgroup estimate in our analysis may reflect measurement-related overestimation rather than a true difference in underlying prevalence.

Studies using subjective refraction also reported a markedly higher prevalence than those using objective methods (63% versus 17%). This difference may reflect patient response bias and examiner variability, particularly among younger children who may have difficulty providing reliable feedback. Objective refraction, especially when combined with cycloplegia, provides more consistent measurements and is less susceptible to accommodative error, making it more appropriate for large-scale prevalence estimation (39).

Regional variation was also observed, with East Malaysia reporting a slightly higher prevalence than Peninsular Malaysia (29% versus 22%). However, this finding should be interpreted cautiously because only three studies from East Malaysia were included, all conducted in Sarawak, compared with 14 studies from Peninsular Malaysia. This limited geographical representation may affect generalisability and may partly explain the observed regional variation.

Although the earliest study included in our review timeline began in 1990, Malaysian research on refractive errors predates this period. Chandran conducted a four-year comparative study of refractive errors among individuals aged 5–65 years in West Malaysia and reported a high distribution of myopia, particularly among Chinese and Malay populations (40).

**Figure 4:**
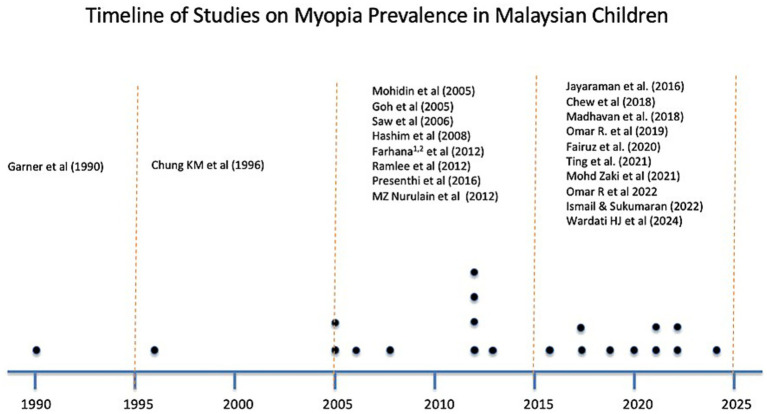
Timeline of included studies according to year of publication.

Similarly, Teoh and Yow examined visual defects and squints among Standard One schoolchildren, reporting refractive errors in 7.1% of children and highlighting divergent squints associated with myopia (5). These early studies show that interest in childhood refractive errors in Malaysia has existed for more than five decades. However, as shown in Figure 4, subsequent research has remained sporadic, geographically uneven, and methodologically diverse. This fragmented evidence base strengthens the rationale for the present review as an evidence-mapping synthesis to guide more standardised national research and policy planning.

### 4.2 Asian and global context

Compared with neighboring countries, Malaysia’s pooled prevalence is lower than that in highly urbanized regions such as Singapore, where school-aged prevalence reaches 50% (41), and parts of China, where rates often surpass 70% among children and adolescents (42). However, it is higher than that in some South Asian populations, such as India and Nepal, where the prevalence in children remains below 15–20% (43–45). These regional differences may reflect a combination of genetic predisposition, environmental exposure, and lifestyle factors, particularly the intensity of near work and reduced outdoor activity associated with urban living (46). Malaysia’s moderate prevalence may, therefore, represent a transitional pattern, with the risk of escalation if urbanization and educational pressures increase.

When situated within global epidemiology, Malaysia’s prevalence mirrors the intermediate levels observed in middle-income countries undergoing rapid urban development. In Europe and North America, childhood myopia prevalence remains relatively low (20–30%) (47), whereas in East Asia, it has reached epidemic levels (46). Therefore, the pooled estimate from Malaysia bridges the gap between high-prevalence East Asian populations and lower-prevalence Western populations, underscoring Malaysia’s vulnerability to following the steep upward trajectory seen in neighboring regions if preventive measures are not prioritized.

### 4.3 Trends and age-related patterns

Temporal subgroup analyses revealed a higher prevalence in studies published before 2005 (35%) than in those conducted after 2015 (24%), although this likely reflects methodological differences rather than a true decline. Age stratification to education level showed a higher prevalence among older children, where preschool 1.0% rose to 30% in primary school, consistent with the natural history of myopia progression during school years (48).

Temporal subgroup analyses revealed a higher pooled prevalence of myopia in studies published before 2005 (35%) than in those conducted after 2015 (24%). This apparent decline likely reflects methodological differences, such as variations in diagnostic criteria, sampling strategies, and the use of cycloplegia, rather than a true reduction in prevalence over time.

Age stratification by educational level further demonstrated a marked increase in prevalence with advancing years of schooling. Our findings showed a rise from 1.0% in preschool-aged children to 30% in those attending primary school, consistent with the natural history of myopia progression during early formal education (48, 49). This pattern supports existing evidence that exposure to intensive near work and reduced outdoor time during schooling years are key drivers of early-onset myopia.

### 4.4 Strengths and limitations

The strengths of this review include a comprehensive search across multiple databases, rigorous subgroup and sensitivity analyses to explore heterogeneity, and the use of established JBI quality appraisal tools to enhance the credibility of the synthesis. Importantly, although the observed heterogeneity limits the precision of a single pooled prevalence estimate, it also highlights the value of this review in identifying key methodological inconsistencies in the Malaysian evidence base and clarifying priorities for future research. Nonetheless, several limitations must be acknowledged. First, substantial heterogeneity (I^2^ = 99.3%) persisted across studies, reflecting variability in diagnostic criteria, refraction technique, sampling strategy, and study design. Second, most included studies used non-cycloplegic refraction, which may overestimate myopia prevalence in children because of residual accommodation. Third, the diagnostic threshold for myopia was not uniform across studies, with definitions ranging from ≤ − 0.25 D to ≤ − 1.00 D, which affects case classification and limits cross-study comparability. Fourth, publication bias cannot be excluded given the small number of large-scale population-based studies, as suggested by the four imputed studies in the trim-and-fill analysis. Fifth, the geographical representativeness of the available evidence was limited: most included studies were conducted in urban areas of Peninsular Malaysia, particularly Kuala Lumpur and surrounding regions, whereas East Malaysia (only three studies, all conducted in Sarawak) and rural populations were under-represented. These constraints limit the extent to which the pooled estimate can be considered a definitive truly national prevalence estimate for Malaysian children. Nevertheless, by quantifying the extent of heterogeneity and showing how prevalence estimates vary according to refraction method, diagnostic threshold, and study population, this review provides a necessary evidence map for researchers, clinicians, and policymakers. Future studies should therefore use standardised cycloplegic refraction, consistent myopia definitions, and stratified sampling across Peninsular Malaysia, Sabah, Sarawak, and rural communities to provide more representative national estimates.

### 4.5 Implications and future research

With one in six Malaysian children estimated to have myopia, the condition represents an emerging burden that may escalate if preventive measures are not implemented. Policies promoting outdoor activity in schools, early vision screening, and parental awareness campaigns are urgently needed (50, 51). Future research should prioritize large-scale, population-based studies that employ standardized definitions (SE ≤ −0.5D) and cycloplegic refraction to ensure accurate prevalence estimates. Longitudinal studies such as National Health and Morbidity Survey (NHMS) are also necessary to track incidence, progression, and associated risk factors in diverse Malaysian subgroups. Ultimately, integrating epidemiological data into national eye health strategies will be crucial to prevent myopia from becoming a major cause of visual disability in Malaysia.

## 5 Conclusion

This systematic review and meta-analysis consolidate fragmented evidence on childhood myopia among Malaysian children aged 4–18 years, but the magnitude of the pooled prevalence depends substantially on measurement method, diagnostic threshold, and adjustment for small-study effects. The unadjusted pooled prevalence was 25.0%; however, the trim-and-fill adjusted estimate of 15.8% and the cycloplegic subgroup estimate of approximately 16% suggest that the clinically confirmed prevalence is likely to lie closer to the lower end of an approximate range of 16 to 25%. The unadjusted 25.0% figure should therefore be used with caution and not be presented in isolation as a definitive national prevalence estimate. When placed within the global context, Malaysia’s prevalence occupies an intermediate position between high-prevalence East Asian populations and lower-prevalence Western nations, indicating a critical window for intervention to prevent escalation to epidemic levels.

Ultimately, in this digital era marked by increasing screen time and reduced outdoor activity, the effective integration of epidemiological insights into national eye health strategies is critical to mitigating the long-term impact of myopia.

## Data Availability

The original contributions presented in the study are included in the article/Supplementary material, further inquiries can be directed to the corresponding author.
