# Supplementary material for: Myopia prevalence in Malaysian children: a systematic review and meta-analysis
Source: Front Public Health. 2026 Jun 25;14:1836591. doi: 10.3389/fpubh.2026.1836591 (PMC13347954; doi:10.3389/fpubh.2026.1836591)
Supplement: Supplementary file 1 [file Supplementary_file_1.DOCX]

**Appendix 1 : Search Strategy and Search Terms**

| **Context** | **MESH** | **Keywords** |
| --- | --- | --- |
| Myopia | Myopia | Myopia  Shortsightedness  Nearsightedness |
| Prevalence | Prevalence | Prevalence  Incidence |
| Country | Malaysia | Malaysia |

For **PubMed**, the search strategy will involve combining various keywords and MeSH terms for myopia, prevalence, and Malaysia. The search will be structured as follows:
(((Myopia* OR Shortsightedness* OR Nearsightedness*) OR ("myopia"[MeSH Terms] OR myopia[Text Word])) AND (((Prevalence* OR Incidence*) OR ("prevalence"[MeSH Terms] OR prevalence[Text Word])) OR ("incidence"[MeSH Terms] OR incidence[Text Word]))) AND ("Malaysia"[Text Word]).

**PubMed search on 28 March 2025**

| **Search number** | **Query** | **Sort By** | **Filters** | **Search Details** | **Results** | **Time** |
| --- | --- | --- | --- | --- | --- | --- |
| **10** | (((Myopia OR Shortsightedness OR Nearsightedness) OR ("myopia"[MeSH Terms] OR myopia[Text Word])) AND (((Prevalence OR Incidence) OR ("prevalence"[MeSH Terms] OR prevalence[Text Word])) OR ("incidence"[MeSH Terms] OR incidence[Text Word]))) AND (Malaysia) |  |  | ("myopia"[MeSH Terms] OR "myopia"[All Fields] OR "myopias"[All Fields] OR "Shortsightedness"[All Fields] OR ("myopia"[MeSH Terms] OR "myopia"[All Fields] OR "nearsightedness"[All Fields]) OR ("myopia"[MeSH Terms] OR "myopia"[Text Word])) AND ("epidemiology"[MeSH Subheading] OR "epidemiology"[All Fields] OR "prevalence"[All Fields] OR "prevalence"[MeSH Terms] OR "prevalance"[All Fields] OR "prevalences"[All Fields] OR "prevalence s"[All Fields] OR "prevalent"[All Fields] OR "prevalently"[All Fields] OR "prevalents"[All Fields] OR ("epidemiology"[MeSH Subheading] OR "epidemiology"[All Fields] OR "incidence"[All Fields] OR "incidence"[MeSH Terms] OR "incidences"[All Fields] OR "incident"[All Fields] OR "incidents"[All Fields]) OR ("prevalence"[MeSH Terms] OR "prevalence"[Text Word]) OR ("incidence"[MeSH Terms] OR "incidence"[Text Word])) AND ("malaysia"[MeSH Terms] OR "malaysia"[All Fields] OR "malaysia s"[All Fields]) | 30 | 02:03:51 |
| **9** | Malaysia |  |  | "malaysia"[MeSH Terms] OR "malaysia"[All Fields] OR "malaysia s"[All Fields] | 111,095 | 02:03:40 |
| **8** | ((Myopia OR Shortsightedness OR Nearsightedness) OR ("myopia"[MeSH Terms] OR myopia[Text Word])) AND (((Prevalence OR Incidence) OR ("prevalence"[MeSH Terms] OR prevalence[Text Word])) OR ("incidence"[MeSH Terms] OR incidence[Text Word])) |  |  | ("myopia"[MeSH Terms] OR "myopia"[All Fields] OR "myopias"[All Fields] OR "Shortsightedness"[All Fields] OR ("myopia"[MeSH Terms] OR "myopia"[All Fields] OR "nearsightedness"[All Fields]) OR ("myopia"[MeSH Terms] OR "myopia"[Text Word])) AND ("epidemiology"[MeSH Subheading] OR "epidemiology"[All Fields] OR "prevalence"[All Fields] OR "prevalence"[MeSH Terms] OR "prevalance"[All Fields] OR "prevalences"[All Fields] OR "prevalence s"[All Fields] OR "prevalent"[All Fields] OR "prevalently"[All Fields] OR "prevalents"[All Fields] OR ("epidemiology"[MeSH Subheading] OR "epidemiology"[All Fields] OR "incidence"[All Fields] OR "incidence"[MeSH Terms] OR "incidences"[All Fields] OR "incident"[All Fields] OR "incidents"[All Fields]) OR ("prevalence"[MeSH Terms] OR "prevalence"[Text Word]) OR ("incidence"[MeSH Terms] OR "incidence"[Text Word])) | 6,298 | 02:03:29 |
| **7** | ((Prevalence OR Incidence) OR ("prevalence"[MeSH Terms] OR prevalence[Text Word])) OR ("incidence"[MeSH Terms] OR incidence[Text Word]) |  |  | "epidemiology"[MeSH Subheading] OR "epidemiology"[All Fields] OR "prevalence"[All Fields] OR "prevalence"[MeSH Terms] OR "prevalance"[All Fields] OR "prevalences"[All Fields] OR "prevalence s"[All Fields] OR "prevalent"[All Fields] OR "prevalently"[All Fields] OR "prevalents"[All Fields] OR "epidemiology"[MeSH Subheading] OR "epidemiology"[All Fields] OR "incidence"[All Fields] OR "incidence"[MeSH Terms] OR "incidences"[All Fields] OR "incident"[All Fields] OR "incidents"[All Fields] OR "prevalence"[MeSH Terms] OR "prevalence"[Text Word] OR "incidence"[MeSH Terms] OR "incidence"[Text Word] | 4,581,713 | 02:02:50 |
| **6** | (Myopia OR Shortsightedness OR Nearsightedness) OR ("myopia"[MeSH Terms] OR myopia[Text Word]) |  |  | "myopia"[MeSH Terms] OR "myopia"[All Fields] OR "myopias"[All Fields] OR "Shortsightedness"[All Fields] OR "myopia"[MeSH Terms] OR "myopia"[All Fields] OR "nearsightedness"[All Fields] OR "myopia"[MeSH Terms] OR "myopia"[Text Word] | 32,846 | 02:02:32 |
| **5** | "incidence"[MeSH Terms] OR incidence[Text Word] |  |  | "incidence"[MeSH Terms] OR "incidence"[Text Word] | 1,126,038 | 02:01:26 |
| **4** | "prevalence"[MeSH Terms] OR prevalence[Text Word] |  |  | "prevalence"[MeSH Terms] OR "prevalence"[Text Word] | 985,365 | 02:01:20 |
| **3** | "myopia"[MeSH Terms] OR myopia[Text Word] |  |  | "myopia"[MeSH Terms] OR "myopia"[Text Word] | 31,331 | 02:00:36 |
| **2** | Prevalence OR Incidence |  |  | "epidemiology"[MeSH Subheading] OR "epidemiology"[All Fields] OR "prevalence"[All Fields] OR "prevalence"[MeSH Terms] OR "prevalance"[All Fields] OR "prevalences"[All Fields] OR "prevalence s"[All Fields] OR "prevalent"[All Fields] OR "prevalently"[All Fields] OR "prevalents"[All Fields] OR "epidemiology"[MeSH Subheading] OR "epidemiology"[All Fields] OR "incidence"[All Fields] OR "incidence"[MeSH Terms] OR "incidences"[All Fields] OR "incident"[All Fields] OR "incidents"[All Fields] | 4,581,713 | 02:00:01 |
| **1** | Myopia OR Shortsightedness OR Nearsightedness |  |  | "myopia"[MeSH Terms] OR "myopia"[All Fields] OR "myopias"[All Fields] OR "Shortsightedness"[All Fields] OR "myopia"[MeSH Terms] OR "myopia"[All Fields] OR "nearsightedness"[All Fields] | 32,846 | 01:59:30 |

**Scopus search on 28 March 2025**

For **Scopus**, we will adjust the search strategy to fit their database requirements. The search will focus on the title, abstract, and keywords using the following structure:
The use of TITLE-ABS-KEY ensures the search targets the most relevant parts of the articles.

(TITLE-ABS-KEY (Myopia* OR Shortsightedness* OR Nearsightedness*) AND TITLE-ABS-KEY (Prevalence* OR Incidence*) AND TITLE-ABS-KEY (Malaysia)).

**Result – 4**

**Cochrane Library search on 28 March 2025**

For Cochrane Library, we will use a simple keyword-based search with the terms “Myopia,” “Shortsightedness,” “Nearsightedness,” “Prevalence,” “Incidence,” and “Malaysia” combined with Boolean operators.

Date Run: 28/03/2025 04:57:34

ID Search Hits

#1 MeSH descriptor: [Myopia] explode all trees 1779

#2 nearsightedness 71

#3 shortsightedness 47

#4 myopia 4042

#5 #1 OR #2 OR #3 OR #4 4061

#6 MeSH descriptor: [Prevalence] explode all trees 7290

#7 MeSH descriptor: [Incidence] explode all trees 14029

#8 burden 37532

#9 prevalence 53082

#10 incidence 166860

#11 #6 OR #7 OR #8 OR #9 OR #10 239152

#12 MeSH descriptor: [Malaysia] explode all trees 514

#13 malaysia 4236

#14 malaysian 846

#15 #12 OR #13 OR #14 4490

#16 #5 AND #11 AND #15 3

All is under review article

**Result = 0**

Publish or Perish software for Google scholar search on 28 March 2025

An additional search was carried out using Google Scholar through the **Publish or Perish** software.

The search follows a similar structure but without truncation or advanced search functionalities, ensuring we capture all relevant research published online. Both strategies aim to comprehensively identify literature related to childhood myopia in Malaysia.

Keyword - (myopia OR Nearsightedness OR shortsightedness) AND (Prevalence OR incidence) AND Malaysia

Maximum number of results setting – 1000

**Result – 999**
