# Supplementary material for: Myopia prevalence in Malaysian children: a systematic review and meta-analysis
Source: Front Public Health. 2026 Jun 25;14:1836591. doi: 10.3389/fpubh.2026.1836591 (PMC13347954; doi:10.3389/fpubh.2026.1836591)

**Appendix 2 : Subgroup forest plots**

Region


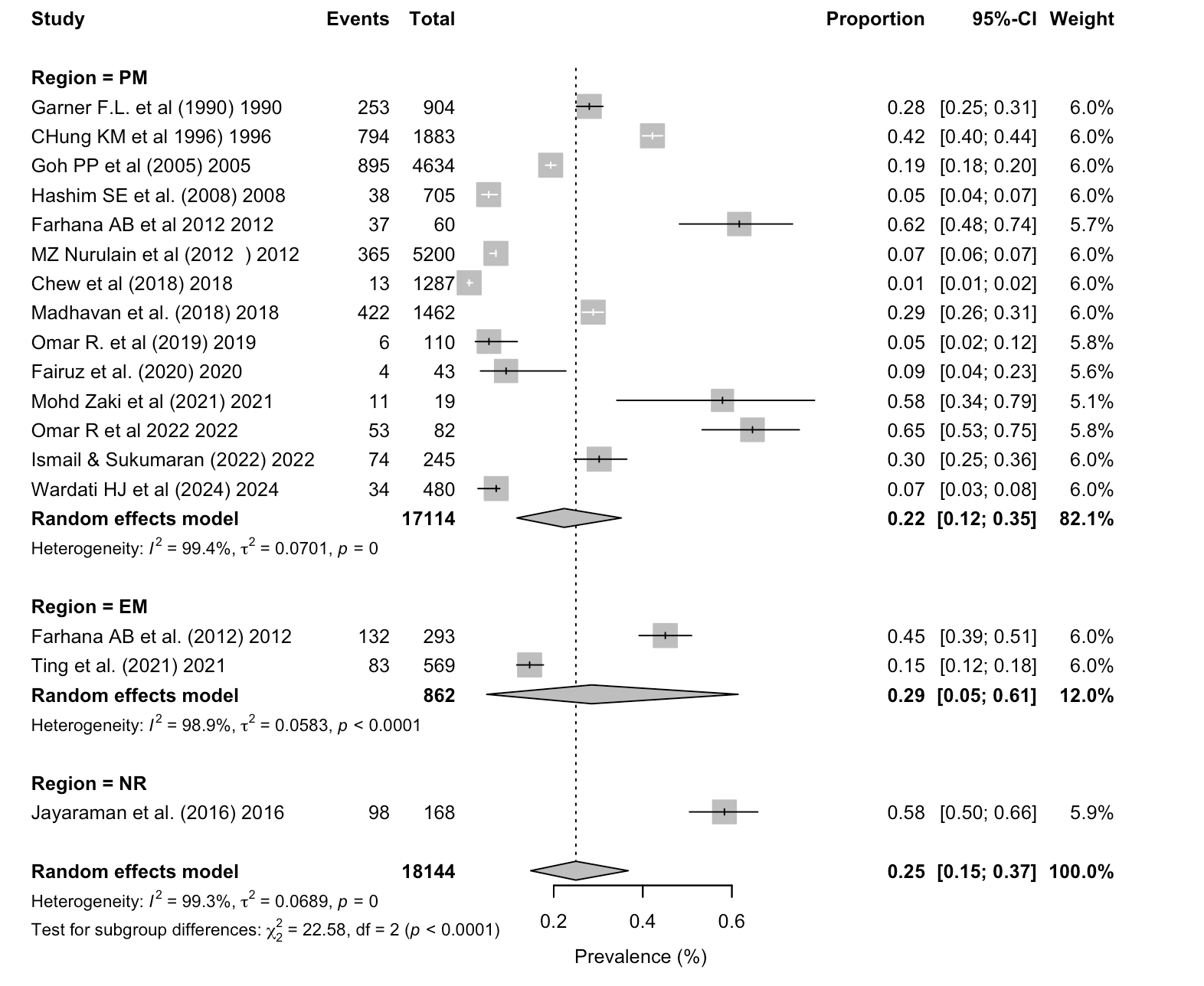


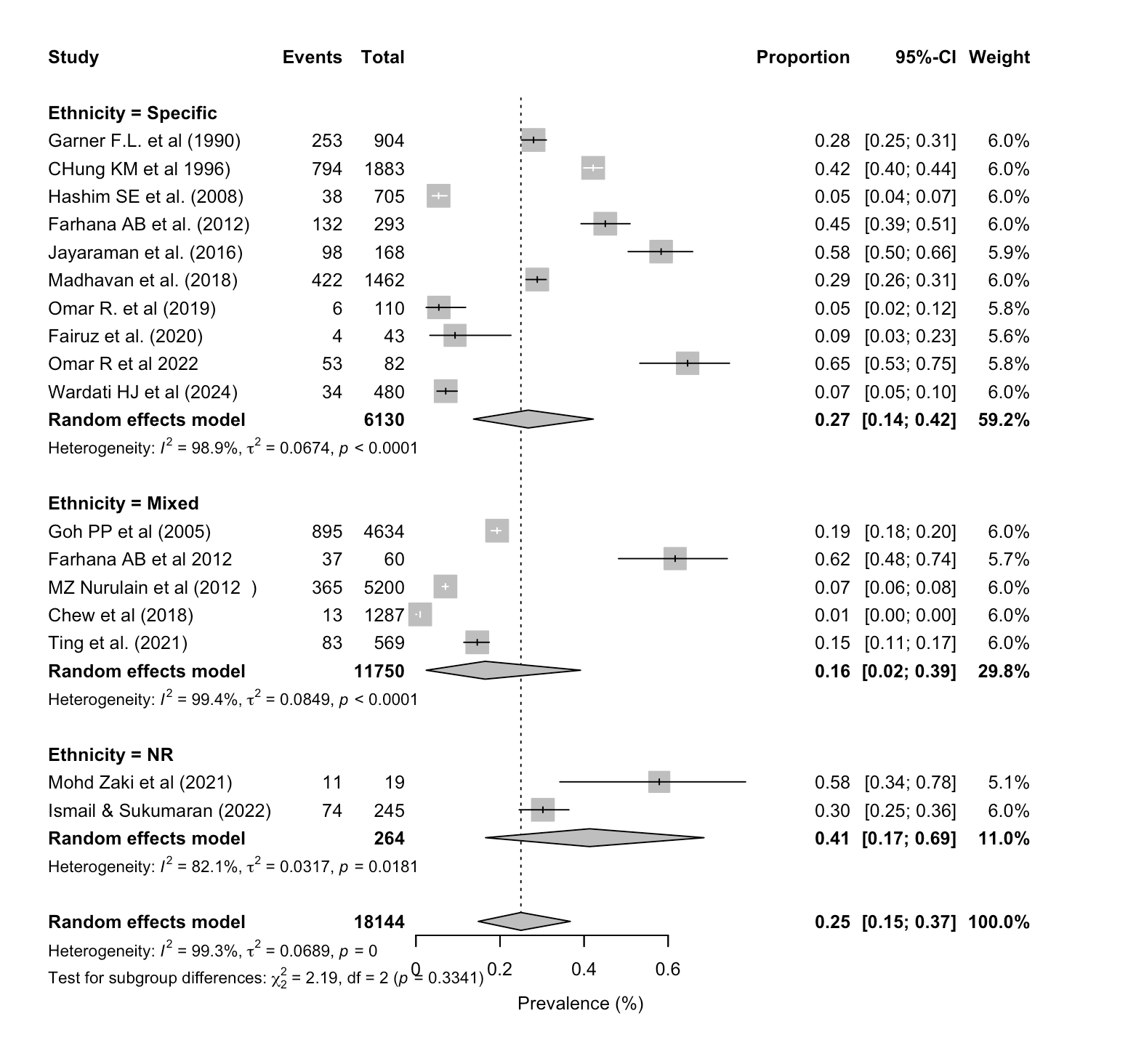
Ethnicity

Education level by age group


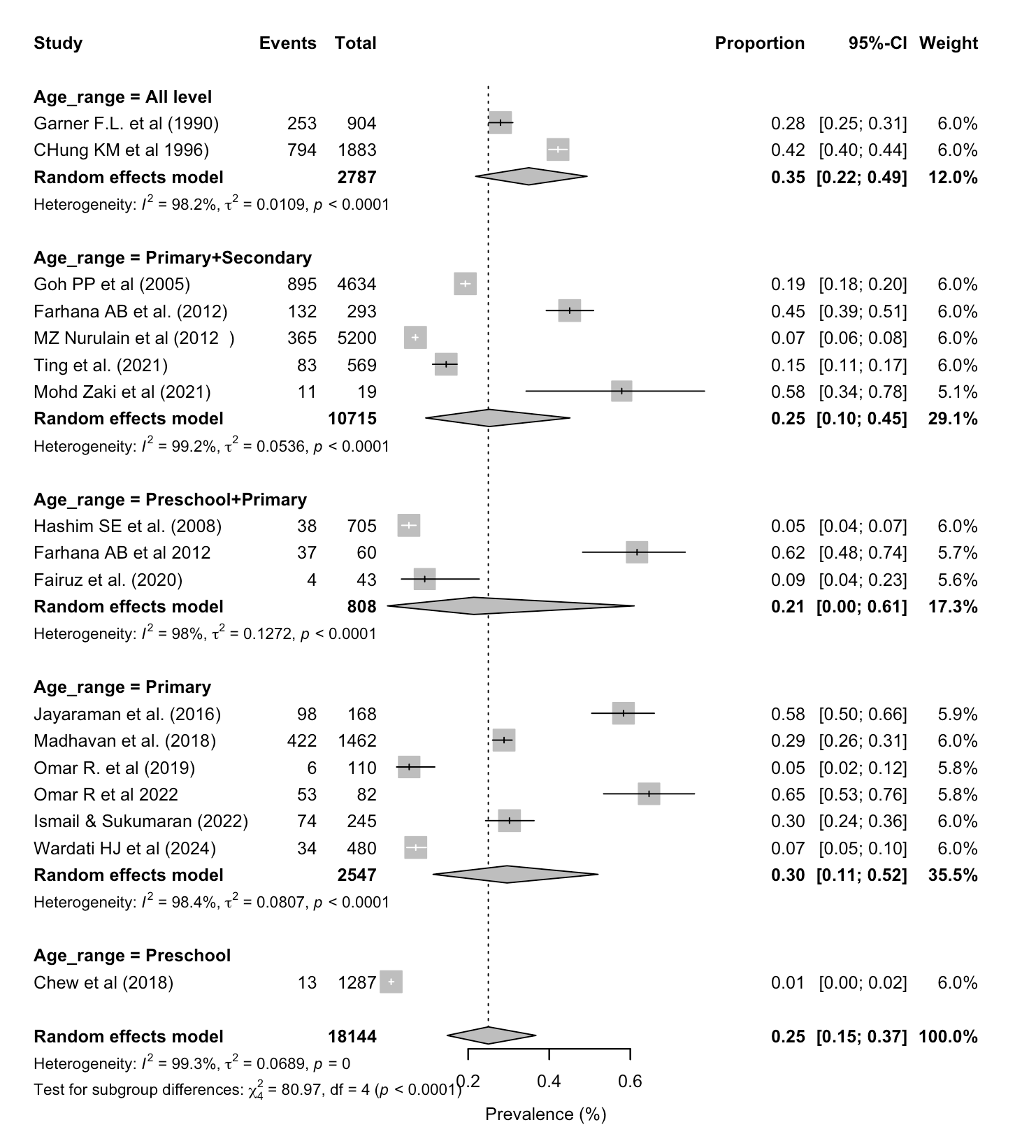


Cycloplegia


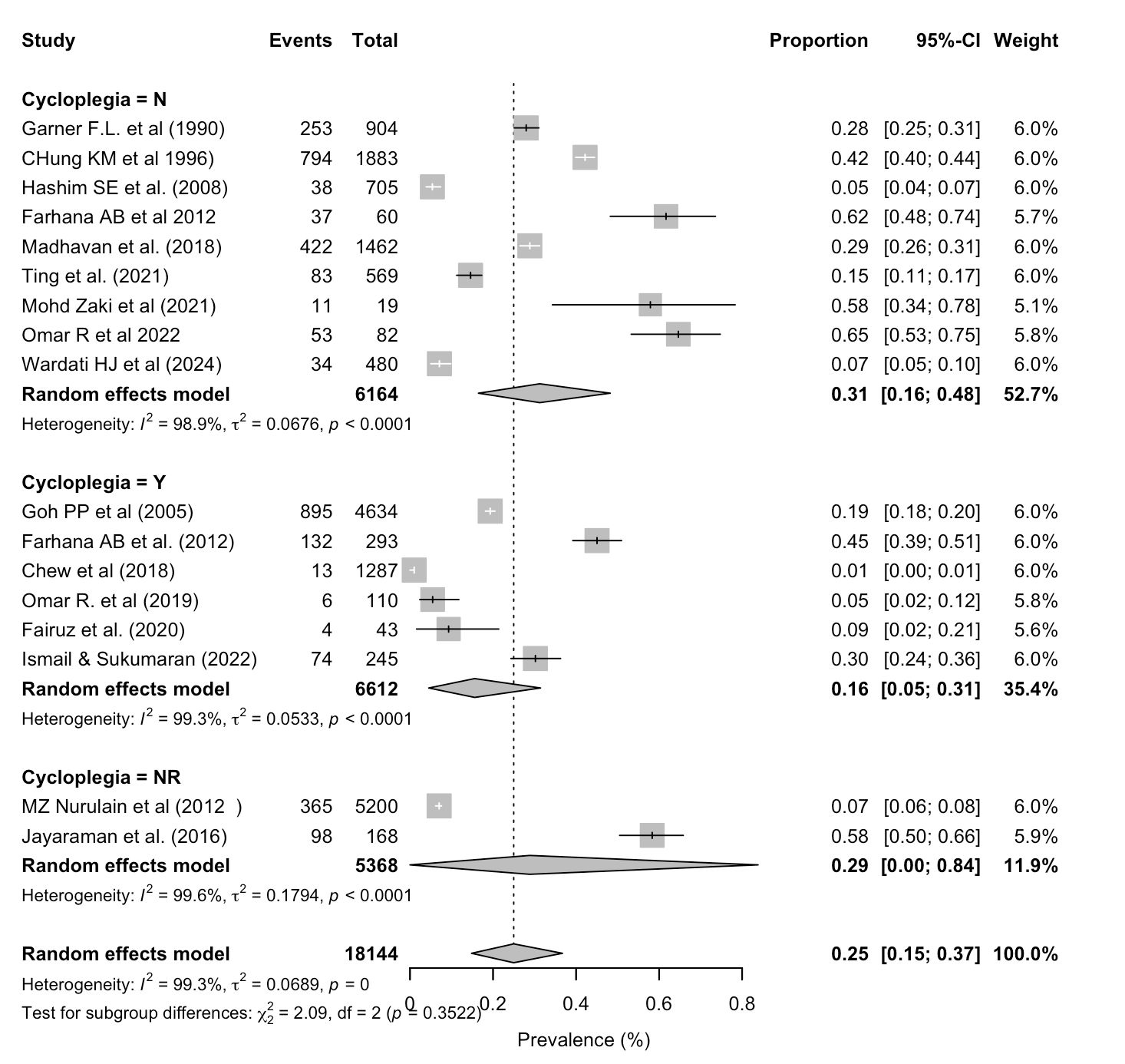


Sample size


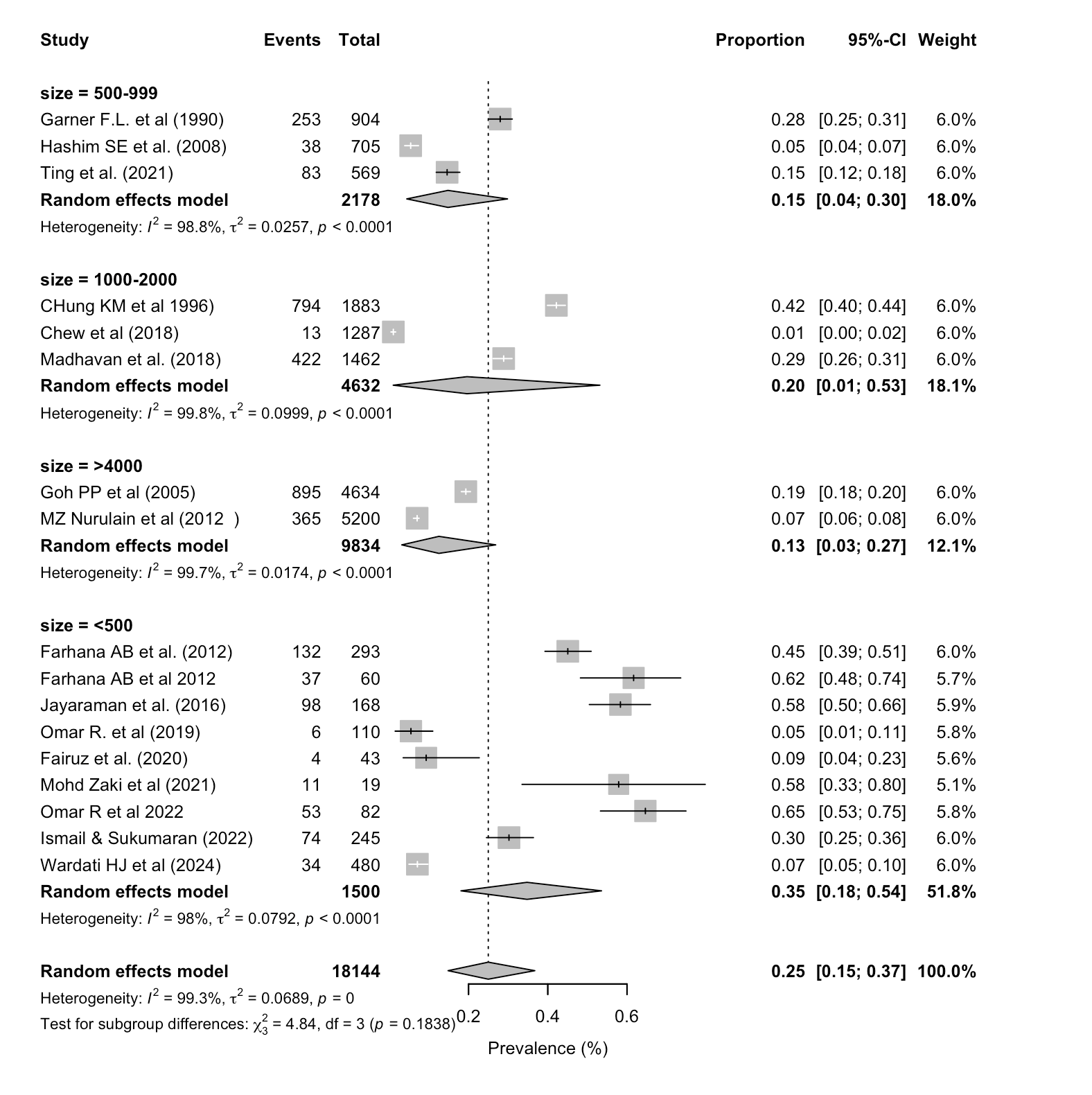


Quality assessment


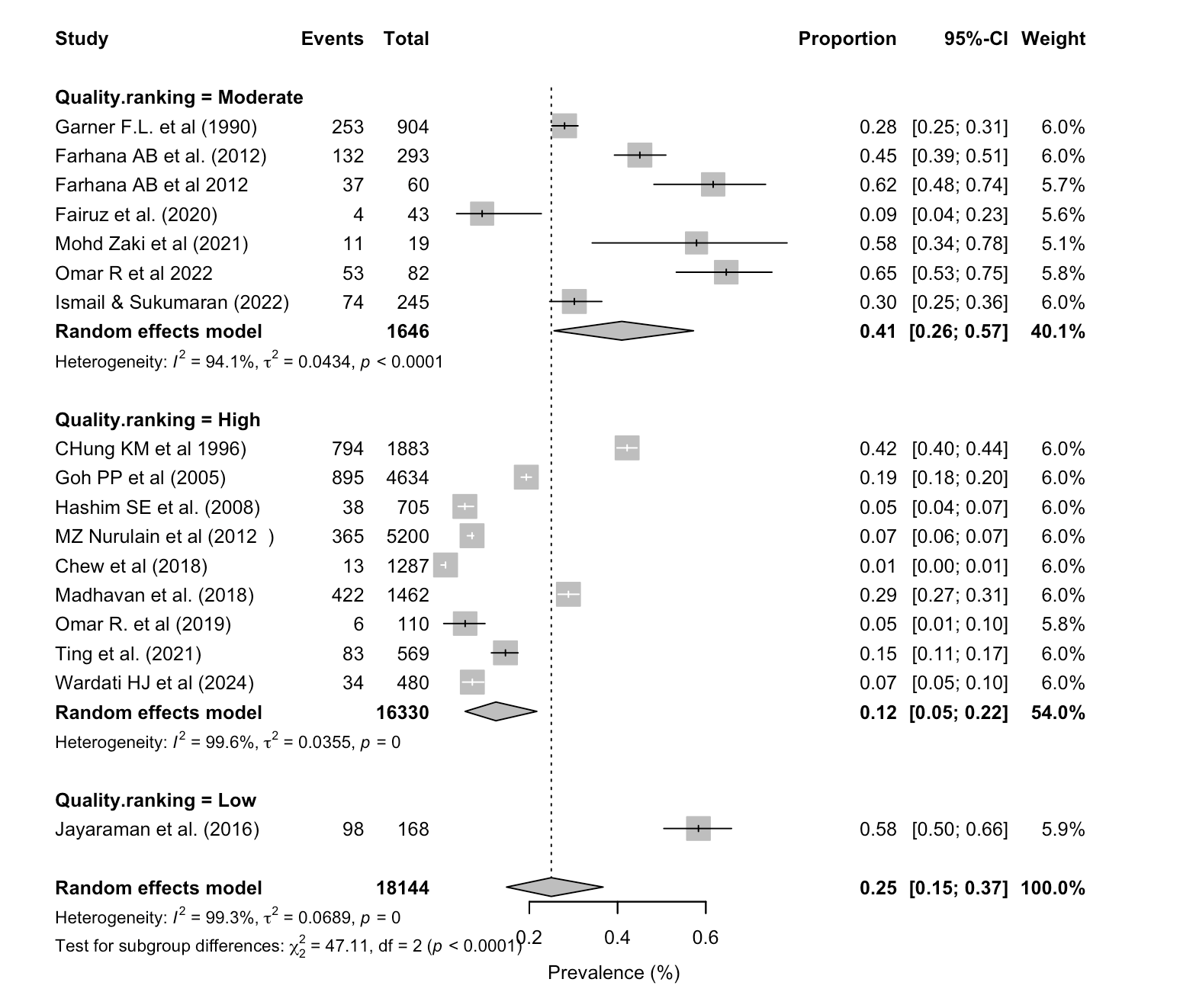


Method


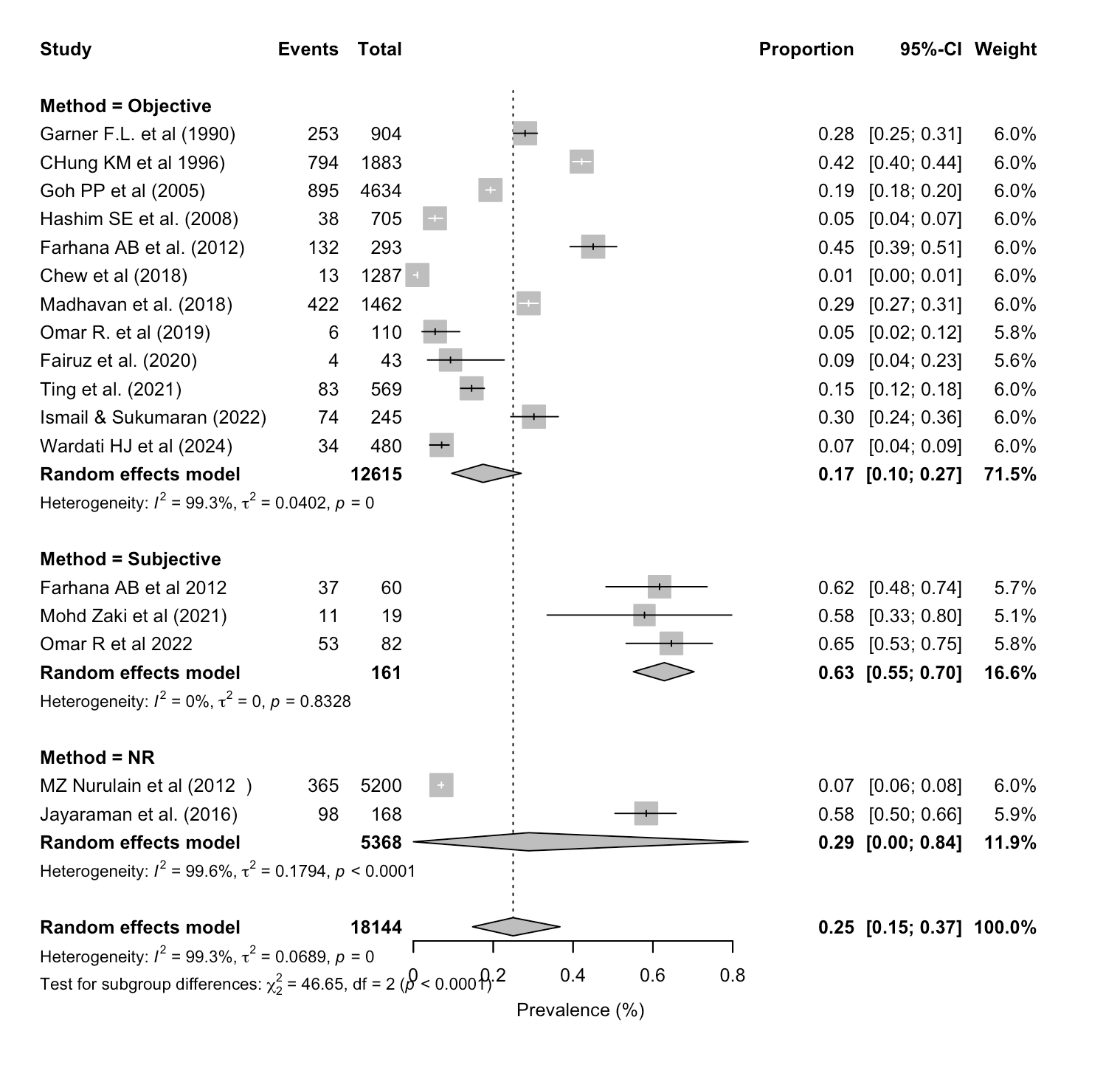


Diopter cut off point


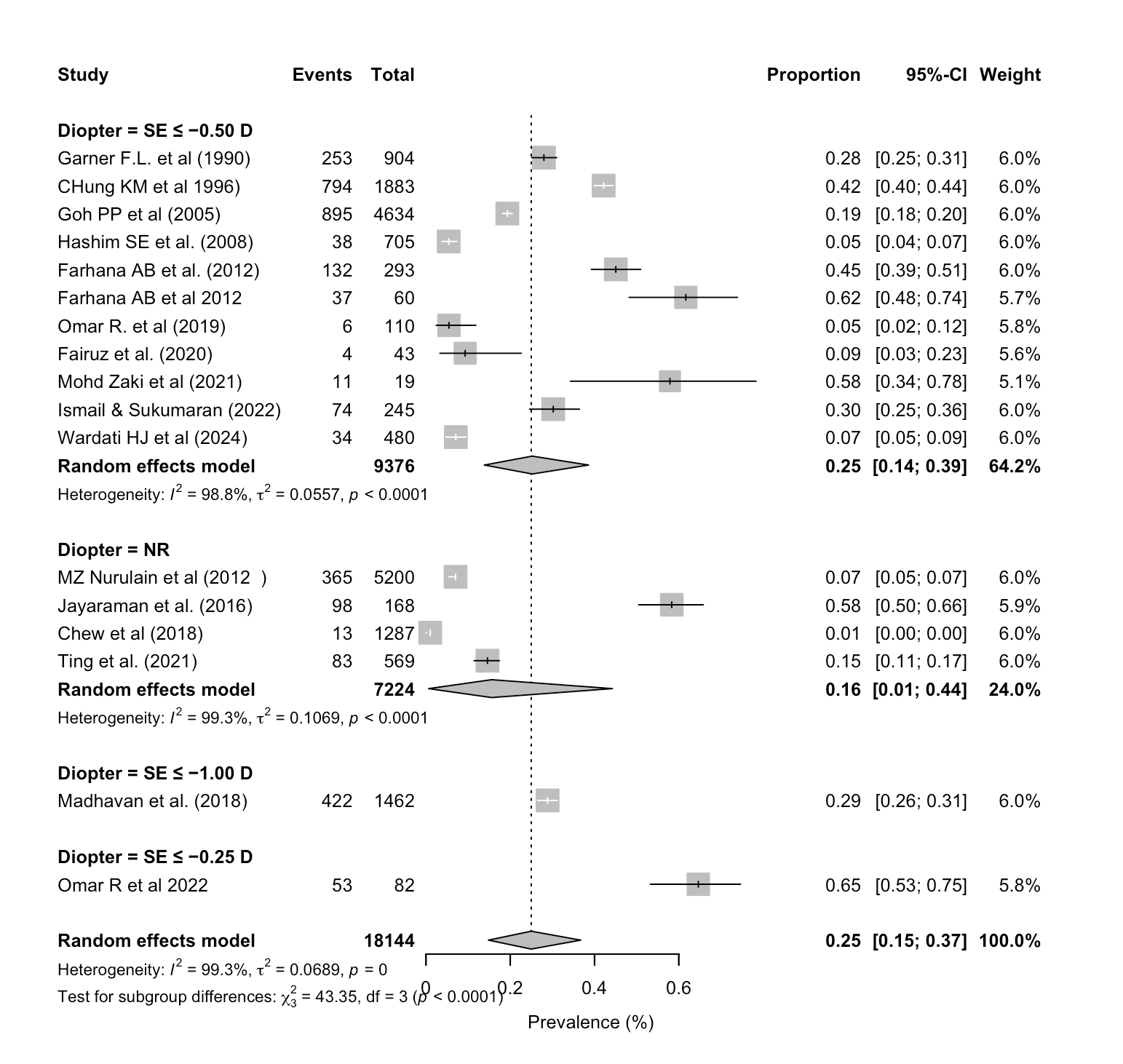


Year of publication


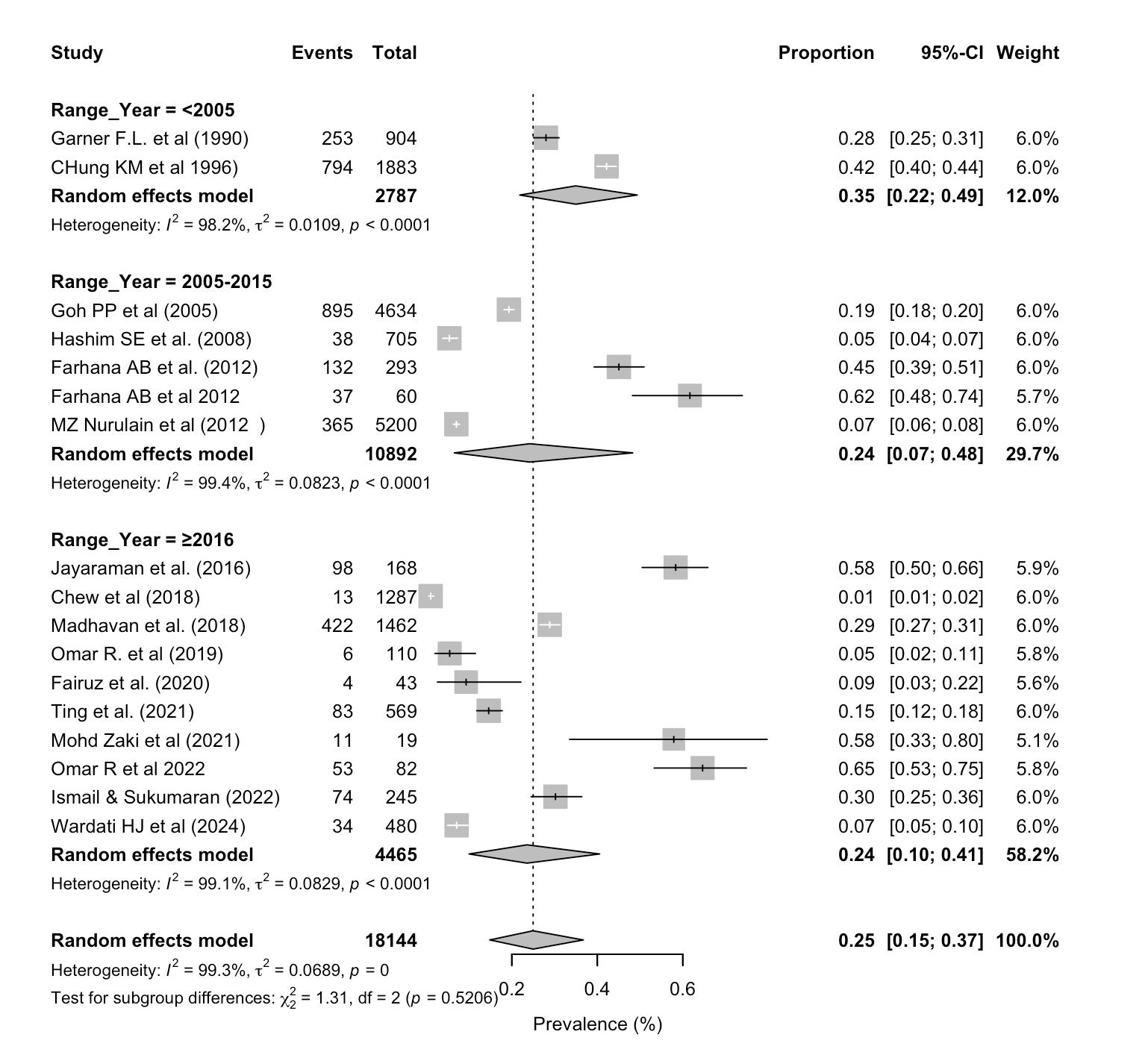


Sample size >500


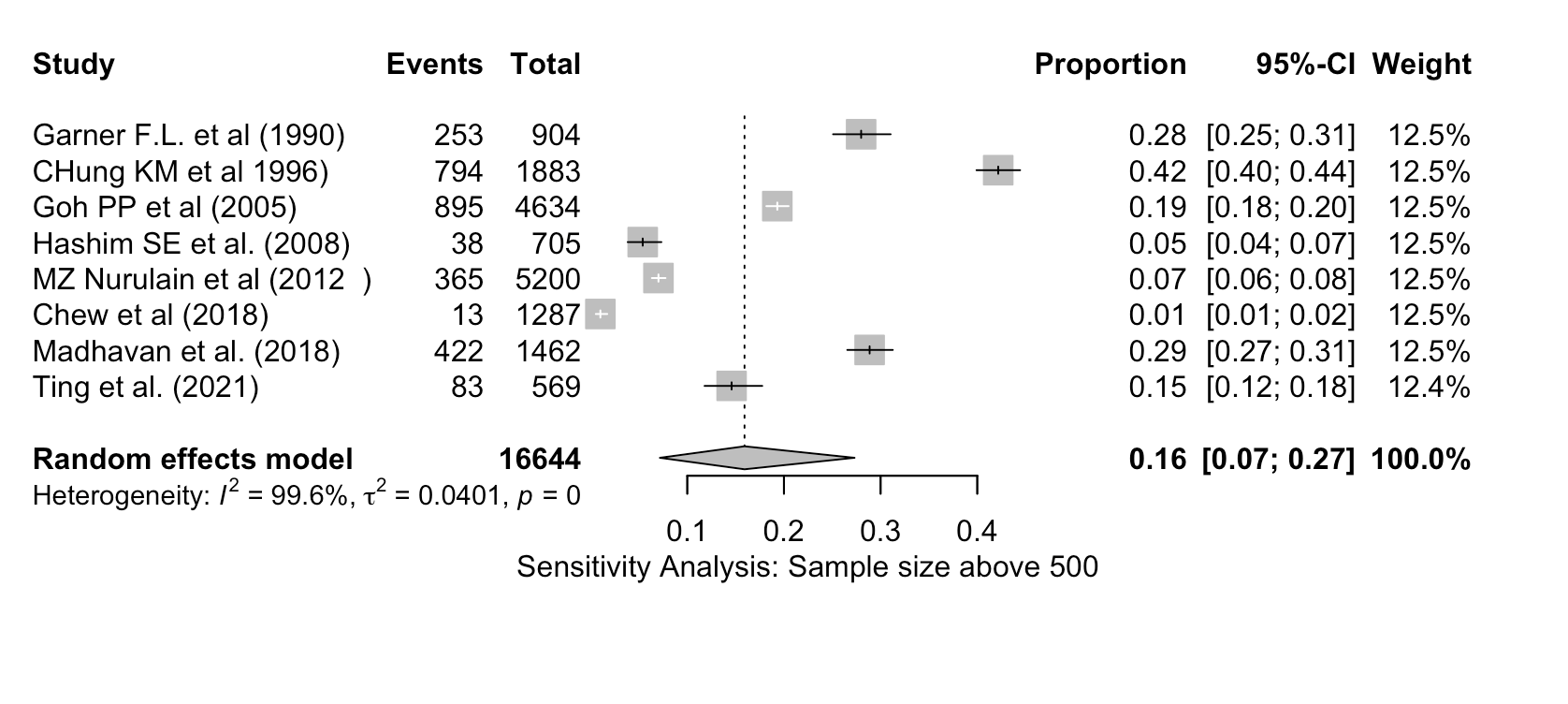


Quality high-moderate


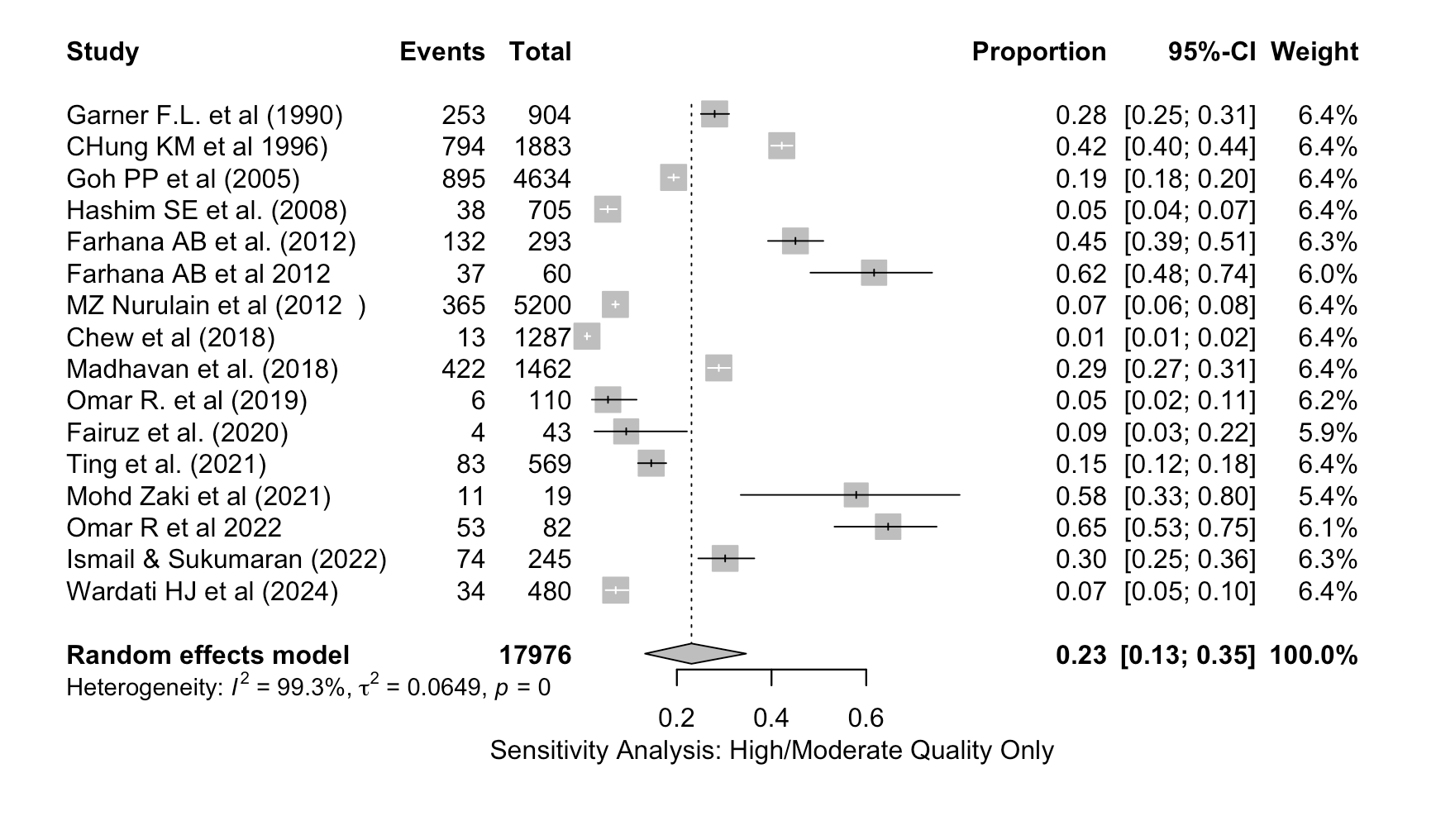

Supplement: Supplementary file 2 [file Supplementary_file_2.DOCX]
