# Supplementary material for: Myopia prevalence in Malaysian children: a systematic review and meta-analysis
Source: Front Public Health. 2026 Jun 25;14:1836591. doi: 10.3389/fpubh.2026.1836591 (PMC13347954; doi:10.3389/fpubh.2026.1836591)
Supplement: Supplementary file 3 [file Supplementary_file_3.DOCX]

| **Domain**  **JBI Prevalence Checklist** | **1.Was the sample frame appropriate to address the target population?** | **2.Were study participants sampled in an appropriate way?** | **3.Was the sample size adequate?** | **4.Were the study subjects and the setting described in detail?** | **5.Was the data analysis conducted with sufficient coverage of the identified sample?** | **6.Were valid methods used for the identification of the condition?** | **7.Was the condition measured in a standard, reliable way for all participants?** | **8.Was there appropriate statistical analysis?** | **9.Was the response rate adequate, and if not, was the low response rate managed appropriately?** | **Overall appraisal** |
| --- | --- | --- | --- | --- | --- | --- | --- | --- | --- | --- |
| ^31^Garner F.L. et al (1990) | N | U | Y | Y | U | Y | Y | N | U | Moderate |
| ^32^Chung KM et al 1996) | N | Y | Y | Y | Y | Y | Y | N | Y | High |
| ^8^Goh PP et al (2005) | Y | Y | Y | Y | Y | Y | Y | Y | Y | High |
| ^33^Hashim SE et al. (2008) | Y | Y | Y | Y | Y | Y | Y | N | Y | High |
| ^9^Farhana AB et al. (2012) | Y | N | N | Y | Y | Y | Y | N | Y | Moderate |
| ^15^Farhana AB et al (2012) | N | N | U | Y | Y | Y | Y | N | U | Moderate |
| ^16^MZ Nurulain et al (2012  )* | Y | Y | Y | Y | Y | Y | Y | N | U | High |
| ^17^Jayaraman et al. (2016) | U | N | Y | N | Y | N | N | N | U | Low |
| ^18^Fiona Min LC et al (2018) | Y | Y | Y | Y | Y | Y | Y | Y | Y | High |
| ^19^Madhavan et al. (2018) | Y | Y | Y | Y | Y | Y | Y | N | Y | High |
| ^21^Omar R. et al (2019) | Y | Y | U | Y | Y | Y | Y | Y | Y | High |
| ^22^Fairuz et al. (2020) | N | U | N | Y | Y | Y | Y | N | N | Moderate |
| ^23^Ting et al. (2021) | Y | Y | Y | Y | Y | Y | Y | Y | N | High |
| ^24^Mohd Zaki et al (2021) | Y | U | Y | Y | U | Y | Y | Y | U | Moderate |
| ^20^Omar R et al 2022 | Y | Y | N | Y | Y | U | N | N | U | Moderate |
| ^25^Ismail & Sukumaran (2022) | Y | N | U | Y | Y | Y | Y | N | U | Moderate |
| ^26^Wardati HJ et al (2024) | N | U | Y | Y | Y | Y | U | Y | Y | High |

**Appendix 3 – JBI assessment**

Y – Yes, N – No, U-unclear, NA- Not applicable, Ranking scale : Low (1-3) , Moderate (4-6), High (7-9), **non-peer reviewed*

*Munn Z, Moola S, Lisy K, Riitano D, Tufanaru C. Methodological guidance for systematic reviews of observational epidemiological studies reporting prevalence and incidence data. Int J Evid Based Healthc. 2015;13(3):147–153.*
